# Effects of Almond- and Olive Oil-Based Docosahexaenoic- and Vitamin E-Enriched Beverage Dietary Supplementation on Inflammation Associated to Exercise and Age

**DOI:** 10.3390/nu8100619

**Published:** 2016-10-09

**Authors:** Xavier Capó, Miquel Martorell, Antoni Sureda, Joan Riera, Franchek Drobnic, Josep Antoni Tur, Antoni Pons

**Affiliations:** 1 Research Groupon Community Nutrition and Oxidative Stress, Science Laboratory of Physical Activity, Department of Fundamental Biology and Health Sciences, University of Balearic Islands, Crtra, Valldemossa, Palma de Mallorca 07122, Illes Balears, Spain; xavier.capo@uib.es (X.C.); martorellpons@gmail.com (M.M.); tosugo@hotmail.com (A.S.); pep.tur@uib.es (J.A.T.); 2 CIBER: CB12/03/30038 Fisiopatología de la Obesidad la Nutrición, CIBEROBN, Instituto de Salud Carlos III (ISCIII), University of Balearic Islands, Palma de Mallorca 07122, Illes Balears, Spain; 3 Departamento de Nutrición y Dietética, Facultad de Farmacia, Universidad de Concepción, Concepción 4070386, Chile; 4 Sports Physiology Department CAR, Barcelona, 08174 Sant Cugat del Vallés, Spain; jriera@car.edu (J.R.); drobnic@car.edu (F.D.)

**Keywords:** inflammation, docosahexaenoic acid, cytokines, physical performance, polyphenol, vitamin E

## Abstract

*n*-3-polyunsaturated fatty acids and polyphenols are potential key factors for the treatment and prevention of chronic inflammation associated to ageing and non-communicable diseases. The aim was to analyse effects of an almond and olive oil beverage enriched with α-tocopherol and docosahexaenoic, exercise and age on inflammatory plasma markers, and immune gene expression in peripheral blood mononuclear cells (PBMCs). Five young and five senior athletes who were supplemented for five weeks with a functional beverage performed a stress test under controlled conditions before and after beverage supplementation. Blood samples were taken immediately before and 1 h after each test. Plasma, erythrocytes and PBMCs were isolated. Beverage supplementation increased plasmatic Tumour Necrosis Factor α (TNFα) levels depending on age and exercise. Exercise increased plasma non esterified fatty acids (NEFAs), soluble Intercellular adhesion molecule 3 (sICAM3) and soluble L-selectin (sL-Selectin), and this increase was attenuated by the supplementation. Exercise increased PGE2 plasma levels in supplemented young and in senior placebo athletes. Exercise increased NFkβ-activated levels in PBMCs, which are primed to a pro-inflammatory response increasing pro-inflammatory genes expression after the exercise mainly in the young group after the supplementation. The functional beverage supplementation to young athletes enhances a pro-inflammatory circulating environment in response to the exercise that was less evident in the senior group.

## 1. Introduction

Functional foods enriched with specific nutrients present in natural foods are a good tool in functional food design. Nuts such as almonds are an important source of essential nutrients, such as arginine, calcium, potassium, niacin, *α*-tocopherol, fibre, monounsaturated fatty acids and polyphenols [1,2]. Nut consumption has been reported to be inversely correlated with the incidence of cardiovascular disease, diabetes and some types of cancer [2,3] and with protective effects against oxidative stress [2,4]. Olive oil is an emblematic ingredient of the Mediterranean diet and is the most important differentiating factor compared to other countries as a source of polyphenols [5]. Olive oil consumption, specifically the extra-virgin variety, is associated with a reduced inflammation and a lower risk of atrial fibrillation and cardiovascular disease and mortality in individuals at high risk for cardiovascular disease [6,7,8]. Supplementing a Mediterranean diet with olive oil or nuts increases the total polyphenol intake, which correlates with higher plasma nitric oxide (NO) and lower systolic and diastolic blood pressure [9]. Supplements containing omega-3 fatty acids, polyphenols, antioxidants and vitamins are widely consumed for better health and athletic achievement [10]. Beneficial effects of dietary supplementation with omega-3 polyunsaturated fatty acids (*n*-3-PUFAs) on exercise performance [11] and on physical activity oxidative balance [12] have been evidenced. Dietary supplementation with *n*-3-PUFAs reduces the inflammatory response against pathogen-associated molecular patterns (PAMPs) in vitro [13,14], although the effects on the immune response associated with intense physical activity are questioned [15,16,17]. The use of functional foods enriched with several nutrients to provide synergic benefits for health and performance is a rising trend [18]. A beverage based on almonds and olive oil, enriched with docosahexaenoic acid (DHA) and vitamin E, could be a good vehicle to supplement athletes’ diet with *n*-3-PUFAs, vitamin E and polyphenols.

Exercise is a good human model for studying the effects of functional food consumption on inflammation. Habitual exercise has been shown to result in an augmented cellular and plasma antioxidant defence system [19,20], reduced lipid peroxidation [21,22] and a protective effect against diseases associated with chronic inflammation [23,24]. Exercise induces a vascular anti-inflammatory response that contributes to counteracting chronic inflammation associated with sedentary habits [25]. Moreover, acute exercise also primes the immune cells for an inflammatory response to PAMPs [13,14].

Ageing is a complex process related to increased inflammation and oxidative stress, with the latter contributing to several age-related changes [26,27]. It has been reported that elderly people are more susceptible than younger people to suffer from oxidative damage in muscles after acute exercise [28]. Age is an additional factor influencing the inflammatory status and demands of *n*-3-PUFA and vitamin E supplementation [29]. *N*-3-PUFAs have now been identified as potential key nutrients that are safe and effective in the treatment and prevention of several adverse consequences of ageing [30]. The effects of supplementing the diet of athletes with functional foods based on almonds enriched with *n*-3-PUFA, olive oil and vitamin E, of plasma markers for inflammation, of markers for immune cell activation, and of the response to acute exercise in young and senior athletes are not known yet.

The aims of this study were to evaluate the effects of both diet supplementation with an almond and olive oil-based beverage enriched with docosahexaenoic and vitamin E, and acute exercise, on erythrocyte fatty acid composition, on plasmatic markers of inflammation and markers of immune cell activation in young and senior athletes.

## 2. Materials and Methods

### 2.1. Subjects and Anthropometric Characteristics

Ten young male taekwondo athletes and eight well-trained male senior athletes related to sport competitions (trainers and sport medical doctors) volunteered to participate in this study. All subjects were informed of its purpose, requirements and possible risks before giving their written consent to take part. Inclusion/exclusion criteria were: Age (18–25 years in young group and 35–57 years in senior group), sex (male), non-smokers, balanced diet, body mass index (19–25 kg/m^2^) and physical activity of 1–2 h daily 5–7 day/week. Before being accepted to participate in the research, each subject underwent a complete medical examination, which included a medical history and resting electrocardiogram (ECG), to prevent any medical problem that would contraindicate the inclusion in the study. The protocol complied with the Declaration of Helsinki for research on human subjects, and was approved by the Ethical Committee for Clinical Research at the Direcció General de l’Esport of the Catalonian Sports Council. The study was registered at ClinicalTrial.gov (NCT02177383). The participants were not acclimatised to heat and the study was conducted in the months of May and June with an average temperature around 18.2 °C and 22.8 °C, respectively. The participants were split into two groups depending on their age. All participants began the nutritional diet trial, but only five young and five senior athletes completed it. The need to participate in sport competitions was the cause to leave the nutritional intervention.

There were no differences in the anthropometric characteristics and physical activity capabilities between the young and senior groups (Table 1).

### 2.2. Beverage Composition

The nutritional intervention consisted of daily supplementation of the diet with one litre of almond and olive oil based functional beverage five days a week for five weeks in place of mineral water that was intake before nutritional intervention in the control situation. The beverage was isotonic (278 mOsm/kg) and made up of 3.0% almond and 0.8% sucrose and the rest was water, flavour, and the added oils and α-tocopherol acetate (vitamin E). Added oils were 0.6% olive oil and 0.2% DHA-S (wt %) (DSM, Columbia, SC, USA). DHA-S is nutritional oil derived from the marine alga *Schizochytrium* sp., a rich source of (DHA) with soy lecithin and rosemary (*Rosmarinus officinalis*) extract as flavour, and tocopherols and ascorbyl palmitate as antioxidants. The procedure for obtaining the beverage was bleaching of the almonds; crushing of the almonds in water; centrifuging of the mixture to eliminate insoluble materials; and the addition of cinnamon and lemon natural flavours, sucrose, vitamin E, and olive oil plus DHA-S. Finally, beverage was sterilized and packed. Functional beverage was elaborated by Liquats Vegetals S.A. (Girona, Spain). The fatty acid composition of the almond beverage enriched with DHA and vitamin E is shown in Table 2. The almond beverage is enriched with olive oil, DHA and a vitamin E contains 2.6% (w %) of fat, 2.85 ± 0.29 mM (51 ± 5 mg/100 mL as l-tyrosine equivalents) of total polyphenols and 4.6 ± 0.3 mg/100 mL of vitamin E (α-tocopherol acetate). The fatty acid content of the beverage is mainly monounsaturated (51.7% ± 5.0%) and polyunsaturated (38.3% ± 4.4%) with a low percentage of saturated fatty acid (9.90% ± 1.15%). The more abundant fatty acids were C18:1 and C18:2 followed by C16:0, C22:6, C18:0 and C22:5, whereas C18:3n3, C18:3n6, C20:2, C20:0 C20:4 and C22:0 were under 1% of total fat content of the functional beverage.

The fatty acid composition of beverage was determined following the same procedure used to determine erythrocyte fatty acid composition as described below. Similarly, polyphenol content of beverage was determined following the same procedure used to determine polyphenol content of plasma, erythrocytes and blood described below. Total fat content of functional beverage was 2.6%, taking into account 60% of fat content of almonds, the olive oil and DHA-S added.

### 2.3. Experimental Procedure

**Figure 1:**
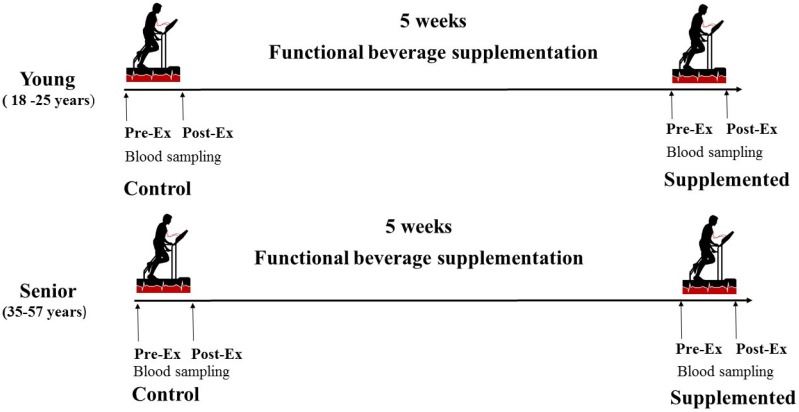
Diagram of the treatment time line.

Athletes performed a stress test in controlled conditions at the beginning of the nutritional intervention and after 5 weeks of beverage supplementation (Figure 1).

Stress test consisted in incremental maximal test until exhaustion on a motorised treadmill (EG2, Vitoria, Spain) to determine their maximal oxygen consumption (VO_2max_) using a computerised metabolic chart (Master Screen CPX, Erich Jaeger, Würzburg, Germany). The velocity corresponding to 60% (V_60_), 70% (V_70_), 80% (V_80_) and 90% (V_90_), of their VO_2max_ was calculated by linear interpolation of data from the maximal exercise test. Subjects arrived at the laboratory at 9:00 a.m. after an overnight fast and having drunk a minimum of 500 cubic centimetre (cc) of water since wakening. Dry nude body weight was measured before and after the stress test after the subjects had emptied their urinary bladder. The subjects equipped with a heart rate transmitter and skin thermistors entered into the climatic chamber set at 30 °C temperature and 70% humidity; after 10 min the baseline core temperature [31], skin temperature and heart rate (HR) values were collected. Subjects continuously ran on the treadmill at the speed of V_60_ for 5 min, V_70_ for 5 min and V_80_ for 5 min for three consecutive bouts with two minutes of recovery between bouts. Finally, the subjects ran at V_90_ until exhaustion, and this time was measured as a quantity of exercise performance. Subjects were required to wear the same clothes and shoes in the two exercise sessions. Water was provided ad libitum in 500 mL bottles at room temperature and the amount of water consumed was measured. The percentage of dehydration was calculated from the weight difference corrected by drinking water during the test. The Polar^®^ heart watch system (Polar Electro Inc., Kempele, Finland) was used to measure basal HR every 5 min during the test and after 5 min of recovery time. A microsample of blood (20 µL) was taken from the ear lobe to measure lactate concentration, at rest at minutes 15, 32, 49 and immediately at the end of the last bout to exhaustion (Dr. Lange^®^, Berlin, Germany). The Borg scale was used to assess subjective perception of effort at Minutes 15, 32, and 49, and after concluding the test [32].

Venous blood samples were obtained from the antecubital vein of participants with vacutainers containing EDTA (ethylenediaminetetraacetic acid) as an anticoagulant for blood count analyses (2 mL), to obtain erythrocytes and plasma (6 mL) and purify peripheral blood mononuclear cells (PBMCs) (6 mL). Venous blood samples were obtained after 12 h, overnight, in fasting conditions (basal sample), and 1 h after finishing training, which is consonant with increased circulating immune cells and significant changes in antioxidant enzyme activities and in markers for oxidative damage.

Erythrocyte fraction was obtained after centrifugation at 900× *g*, 30 min, 4 °C. Then, erythrocytes were washed with phosphate buffered saline (PBS), centrifuged at 900× *g*, 20 min, 4 °C and lysed with distilled water at the initial blood volume. Cell lysates were stored at −80 °C until biochemical analyses thereof.

PBMCs were obtained following a method previously described [33]. Blood was carefully introduced on Ficoll in a proportion of 1.5:1 and was then centrifuged at 900× *g*, at 4 °C for 30 min. The PBMCs layer was carefully removed. The plasma and the Ficoll phases were discarded. The PBMCs slurry was then washed twice with PBS and centrifuged for 10 min at 1000× *g*, 4 °C. This process was performed in triplicate, with one of the samples used to obtain RNA, and another being lysed with distilled water. Cell lysates were stored at −80 °C until biochemical analyses were performed.

### 2.4. Fatty Acid Determination

Erythrocyte and beverage fatty acids were extracted in duplicate with chloroform/methanol (2:1 v/v) by a modified method of Folch [12,34], containing 0.01% butylated hydroxyanisole as antioxidant and 20 µL of n-heptadecanoic acid (15 mM) as the internal standard. The resultant organic phase was evaporated under a nitrogen stream at 55 °C. The dry residue was dissolved in 225 µL of n-hexane and 25 µL of Meth-Prep™ II (Grace Davison Discovery Sciences, Columbia, MD, USA) and the derivatization reagent was added. The gas chromatograph was an Agilent 5890 model (Agilent Technologies, Santa Clara, CA, USA) with a flame ionization detector (FID) and the column was a Supelcowax^®^ 10 Capillary GC column, 30 m × 0.53 mm × 0.50 µm (Supelco, Bellefonte, PA, USA).

### 2.5. Polyphenols Determination

Total polyphenol content of the functional beverage was determined through the Folin–Ciocalteau method [35] in the supernatants of deproteinized samples with cold acetone (1:1.2) using l-tyrosine as standard. The results are expressed as mmols of l-tyrosine/L.

### 2.6. Cytokine, Eicosanoids and Adhesion Molecules Determination

Prostaglandin E1 (PGE_1_) and Prostaglandin E2 (PGE_2_) were measured in plasma using ELISA kits (Enzo Life Sciences^®^, Farmingdale, NY, USA). Intra-assay and inter-assay reproducibility for PGE_1_ were lower than 10% and 12%, respectively, while intra-assay and inter-assay reproducibility for PGE_2_ were lower than 6% in both cases.

Lipoxin A4 was measure in plasma using ELISA kit (CUSABIO^®^, Baltimore, MD, USA). Intra-assay and inter-assay reproducibility were lower than 8% and 10% respectively.

Interleukin-6 (IL-6) and Tumour Necrosis Factor α (TNFα) were measured in plasma using ELISA kits (DIACLONE^®^, Besançon cedex, France). Intra-assay and inter-assay reproducibility for IL-6 were calculated to be 3.3% and 9.1% respectively, while intra-assay and inter-assay reproducibility for TNFα were calculated to be 4.4% and 9.0% respectively.

sL-Selectin and sICAM-3 were measured in plasma using ELISA kits (DIACLONE^®^, Besançon cedex, France). Intra-assay and inter-assay reproducibility for sL-Selectin were calculated to be 4.6% and 3.22% respectively, while intra-assay and inter-assay reproducibility for sICAM-3 were calculated to be 3.49% and 1.99%, respectively.

### 2.7. Non Esterified Fatty Acids Determination

Non esterified fatty acids were determinate in plasma using an enzymatic kit (Wako^®^) based on the specificity of acyl-CoA synthetase for the free fatty acids.

### 2.8. Nuclear Factor κβ (NFκβ) Activation Quantification

An isolated suspension of PBMCs was subjected to whole-cell protein extraction for the determination of NFκβ p50 activation, which was performed using an ELISA method according to the manufacturer’s instructions TransAM NF-kB p50 Chemi (Active Motif^®^). Briefly, the primary antibody used to detect NFκβ recognizes an epitope on p50 that is accessible only when NFκβ is activated and bound to its DNA target.

### 2.9. Gene Expression

Toll like receptor 2 (TLR2), Toll Like receptor 4 (TLR4), Nfκβ, Cyclooxygenase 2 (COX2), 5 Lipoxygenase (5 LOX), 15 Lipoxygenase 2 (15 LOX 2), Interleukin 1β (IL1β), Interleukin-8 (IL-8), Tumour Necrosis Factor (TNFα), Interleukin-10 (IL-10), Interleukin-15 (IL-15) and Heat Shock Protein 70 (HSP70) mRNA expression was determined by multiplex real-time PCR based on incorporation of a fluorescent reporter dye and using human 18S rRNA as reference. For this purpose, total RNA was isolated from PBMCs by Tripure extraction (Roche Diagnostics, Germany). RNA (1 µg) from each sample was reverse transcribed using 50 U of Expand Reverse Transcriptase (Roche Diagnostics, Germany) and 20 pmol oligo (dT) for 60 min at 37 °C in a 10 µL final volume, according to manufacturer instructions. The resulting cDNA (2.5 µL) was amplified using the LightCyclerFastStart DNA MasterPLUS SYBR Green I kit (Roche Diagnostics, Germany). Amplification was performed at 55 °C and 45 cycles. The relative quantification was performed by standard calculations considering 2^(ΔΔCt)^. Antioxidant enzyme levels before and after the season were normalized to the invariant control 18S rRNA. mRNAlevels at basal young control group were arbitrarily referred to as 1. Primers used are listed in Table 3.

### 2.10. Statistical Analysis

Statistical analysis was carried out using the Statistical Package for Social Sciences (SPSS v.21.0 for Windows). Results are expressed as mean ± SEM and *p* < 0.05 was considered statistically significant. A Kolmogorov–Smirnov test was previously applied to assess the normal distribution of the data. The statistical significance of the data was assessed by a three-way analysis of variance (ANOVA). Bonferroni test was used in order to make a multiple comparison. The statistical factors analysed were beverage supplementation (S), ageing (A) and exercise (E). For the sets of data where there was a significant S × E × A, S × E, S × A, and A × E interactions were tested by the ANOVA one-way test.

## 3. Results

### 3.1. Effects on Exercise Performance Parameters

Neither functional beverage diet supplementation nor age altered the exercise performance parameters (Table 4). Maximum exercise tests increased core and skin temperature in a similar way in all groups and situations until a maximum core temperature of about 39.4 °C and a maximum skin temperature of about 34.8 °C were attained. Heat storage during the maximum exercise test was similar in all groups and situations. The heart rate attained during the exercise test was about 97.4% of maximum heart rate and similar in all groups and situations. The physiological strain index attained during the exercise test was about 9.88 and similar in all groups and situations, which points to very high heat stress induced by the exercise test. Similarly, the Borg index of fatigue also indicates very fatiguing exercise with no influence from the functional beverage on this perception, although the senior group did perceive a significantly lower fatigue sensation than the younger group during the exercise test. The time spent running at 90% VO_2max_ until exhaustion was similar in the young and senior groups, regardless of the control or functional beverage supplemented situation. The maximum blood lactate level, water intake and weight loss during the exercise tests were similar in all groups and situations. In summary, the exercise test was highly fatiguing for athletes showing a very high heat stress who attained the anaerobic exercise phase with high core and skin temperature values and a moderate weight loss not influenced by either age or supplementation.

### 3.2. Effects on Fatty Acids Composition

**Figure 2:**
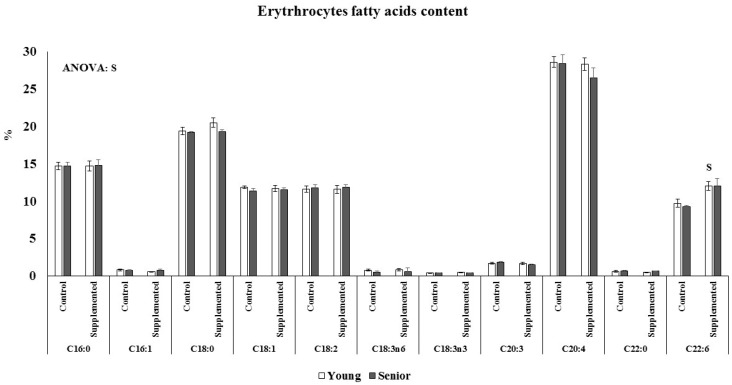
Effects of age and beverage supplementation on erythrocytes fatty acids composition. Results are the mean ± SEM. Statistical analysis: Two-way ANOVA, *p* < 0.05. S, supplementation effect; A, age effect, S × A, interaction between supplementation and age effects. * Indicates significant differences between the dietary control and functional beverage dietary supplementation plasma levels; ^$^ indicates significant differences between Young and Senior groups. When interaction exists between different statistical factors, different letters reveal significant differences.

**Figure 3:**
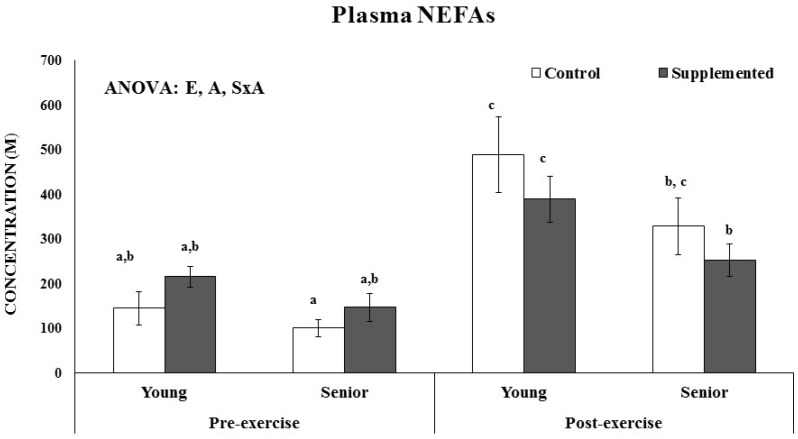
Age, dietary beverage supplementation and acute exercise effects on NEFAs plasma levels. Results are the mean ± SEM. Statistical analysis: Three-way ANOVA, *p* < 0.05. S, supplementation effect; A, age effect, E, exercise effect, S × A, interaction between supplementation and age effects, S × E, interaction between supplementation and exercise effects, E × A, interaction between exercise and age effects, A × E × S, interaction between three factors effects. S, A, E, S × A, E × A, S × E or A × E × S indicates a significant effect of each statistical factor. * Indicates significant differences between the dietary control and functional beverage dietary supplementation plasma levels; # indicates significant differences between pre-exercise and post-exercise plasma levels; ^$^ indicates significant differences between Young and Senior groups. When interaction exists between different statistical factors, different letters reveal significant differences.

Age and beverage supplementation altered the fatty acid composition of erythrocytes (Figure 2). No age or supplementation effect was observed in the percentage of C16, C16:1, C18, C18:1, C18:2, C18:3n6, C18:3n3, C20:3, C20:4, and C22:0 of erythrocytes. The percentage of C22:6 was significantly higher after dietary supplementation with the functional beverage than in the control situation in both the young and senior groups. The nutritional intervention with one litre of the functional beverage for five days a week was followed by all participants and was effective at enriching erythrocytes with DHA. The plasmatic NEFAs concentration was influenced by acute exercise and age, (Figure 3). The young athletes evidenced significant higher plasma NEFAs after acute exercise, both in the control and experimental situations, whereas in the senior group, the increase was only significant in the control situation. Furthermore, an interaction between supplementation and exercise was observed, resulting in an attenuated response in the supplemented situation respect to the control.

### 3.3. Effects on Inflammatory, Heat Stress and Immune Priming Response Markers

The effects of age, acute exercise and dietary functional beverage supplementation on plasmatic markers of inflammation and heat stress and on inflammatory priming of PBMCs are shown in Table 5. The functional beverage significantly influenced the immune markers: sL-selectin, sICAM3 and TNFα, whereas age influenced sICAM3, and acute exercise influenced lipoxin, PGE2 and the activation of NFkβ in PBMCs. Acute exercise influenced adhesion molecules sL-selectin and sICAM3 plasma levels, depending on the functional beverage dietary supplementation, and it also influenced cytokines IL-6 and TNFα plasma levels in an age- and supplementation-dependent manner. Activated NFkβ levels in PBMCs were significantly higher after acute exercise in both the young and senior groups, mainly after the functional beverage dietary supplementation.

The supplementation with the functional beverage significantly decreased sL-Selectin, aside from the fact that there is an existing interaction between exercise and supplementation; in this sense, acute exercise significantly increased sL-selectin plasma levels mainly in the young group, whereas dietary functional beverage supplementation eliminates this exercise effect and sL-selectin levels post-exercise were similar to pre-exercise plasma levels.

Acute exercise significantly increased sICAM3 plasma levels only in the young group, whereas dietary functional beverage supplementation eliminates this exercise effect and sICAM-3 plasma levels post-exercise for the young group were three times lower than pre-exercise ones. Neither acute exercise nor functional beverage dietary supplementation influenced sICAM3 plasma levels in the senior group. These different patterns of change in the young and senior groups were reflected in pre-exercise and post-exercise sICAM3 plasma levels that were, significantly, about three times lower in the young group than in the senior group after supplementation, but not in the control situation.

No impact from supplementation, acute exercise or age was observed on HSP70 or PGE1 plasma levels.

Acute exercise influenced the plasma levels of lipoxin and PGE2. Exercise tended to decrease lipoxin plasma levels, mainly in the senior group after functional beverage supplementation. In turn, exercise significantly increased PGE2 plasma in the young group after functional beverage supplementation and in the control dietary situation for the senior group.

The effects of the functional beverage supplementation on IL-6 and TNFα plasma levels depended on age and exercise. Plasmatic IL-6 was maintained at the control level in all conditions in the senior group. However, post-exercise plasma levels in the young group after dietary functional beverage supplementation were significantly higher respect to pre-exercise and control levels. Functional beverage supplementation enhanced IL-6 plasma levels in response to exercise in young athletes but not in senior athletes. Dietary functional beverage supplementation also increased plasmatic TNFα levels depending on age and exercise. Pre-exercise plasma TNFα levels were significantly higher after supplementation compared to control in the young group, and the post-exercise TNFα plasma levels in the senior group were significantly higher than in control. Plasma TNFα remained at the same level regardless of the age or exercise situation in control.

### 3.4. Effects on Inflammatory Genes Expression in PBMCs

Neither dietary functional beverage supplementation nor exercise or age influenced the gene expression of TLR4, NFκβ 5 LOX, IL-10, IL-15, HSP72 in PBMCs (Table 6). The supplementation significantly influenced TNFα gene expression in PBMCs, being significantly higher after dietary supplementation than in control for the young group in a pre-exercise situation. Functional beverage supplementation and age significantly increased the 15LOX2 gene expression; 15LOX2 gene expression in the senior group was significantly higher than in the young group, both in pre-exercise and post-exercise situations after dietary supplementation with the functional beverage, whereas no differences were seen in 15LOX2 values in the control situation. Similarly, COX2, IL1β and IL-8 gene expression were influenced by an interaction between dietary functional beverage supplementation and age. The expression of IL1β and IL-8 was enhanced in PBMCs after dietary functional beverage supplementation in the young group, mainly post-exercise, but not in the senior group.

Furthermore, an interaction between acute exercise and age was detected on TLR2 gene expression.

## 4. Discussion

The composition of the functional beverage enables dietary supplementation with the omega 3-fatty acid DHA, vitamin E and polyphenols. The daily intake of one litre of the beverage, five days a week, represents daily supplementation with an average of 18.6 g/day fat (of which 820 mg/day corresponds to DHA), 32.6 mg/day of vitamin E, and 36.4 mg/day of polyphenols. The fat intake with the functional beverage represents about 17% of recommended fat consumption for the general population [37]; however, the DHA and vitamin E intake with the same amount of the functional beverage are about three times higher than Recommended Dietary Allowances (RDA) for the general population. The dietary recommendation of vitamin E for active athletes is based on the daily energy consumed [38]; thus, the vitamin E content in the functional beverage would supply the vitamin E requirements for active athletes expending the energy content amount in the functional beverage itself. In addition, the fatty acid content of the beverage is mainly monounsaturated (51.7% ± 5.0%) and polyunsaturated (38.3% ± 4.4%) with a low percentage of saturated fatty acid (9.90% ± 1.15%). In this light, its consumption could contribute to a shift towards a more unsaturated plasma and cell membrane fatty acid profile. Accordingly, the fatty acid composition of erythrocytes is more unsaturated, mainly due to C22:6 after supplementation. The functional beverage also supplements the diet with polyphenols. The polyphenol content in the beverage is similar to the supplied by an orange juice, the consumption of which decreases basal oxidative damage in the elderly [39].

The exercise test undertaken was highly fatiguing and represents a very high heat stress for athletes, so much so that they attained an anaerobic exercise phase with high core and skin temperature values and moderate weight loss. Neither age nor dietary functional beverage supplementation influenced exercise performance parameters, including no influence from time at 90% VO_2max_ spent to exhaustion. The null influence of the functional beverage intake on exercise performance is in accordance with the null influence of vitamin E and C and polyphenol diet supplementation on the VO_2max_ values for athletes [40]. In turn, the high fat content of the beverage has no negative influence on exercise performance, in contrast to other studies where fat intake reduced physical performance [41]. It does, however, contrast with the positive impact of omega-3 fatty acid consumption on physical performance as described in other studies [42,43]. The exercise test conditions were designed to induce an immune response priming neutrophils and PBMCs to an inflammatory response [14,44], although plasmatic markers of inflammation such as IL-6, IL-10, TNFα, IL1β, etc. point to a more post-exercise anti-inflammatory condition.

## 5. Effects on Inflammatory Markers

Dietary fat could influence inflammatory status [45]. The anti-inflammatory effects of omega-3 fatty acids have long been reported [13,14,46] whereas saturated fatty acids have been linked to inflammation [46]. It has also been demonstrated that almond intake could decrease some inflammatory markers such as protein C reactive and E-selectin, although no effects on IL-6 have been observed yet [47]. It has been pointed out that exercise and ageing influence the degree of inflammation [48,49,50]. The present results showed that exercise, regardless of age or functional beverage supplementation, activates NFκβ in PBMCs that drives the cell to gene expression of pro-inflammatory cytokines, anti-oxidant enzymes and other genes [51,52]. The plasma levels of sL-selectin, sICAM3, Lipoxin, IL-6, TNFα, PGE2 are influenced by exercise [24,49], although their response to exercise are age and beverage dependent. It has been stated that an induction of biosynthesis of pro-inflammatory prostaglandins, leukotrienes and inflammation pro-resolving mediators occurs during the early hours (1–2 h) of post-exercise recovery [53]. Similarly, increased IL-6, TNFα and PGE2 plasma levels after intense exercise have been noted [13,23,54,55], although this response is age- and functional beverage supplementation-dependent. IL-6 increased after exercise indietary supplemented young athletes; PGE2 increased after exercise both in dietary supplemented young athletes and in control senior athletes; the TNFα post-exercise levels of dietary supplemented seniors are higher in than in control senior athletes. TNFα and IL-6 are considered pro-inflammatory cytokines and PGE2 a pro-inflammatory prostaglandin [23,54]. However, it has been suggested that IL-6 exerts anti-inflammatory actions via traditional signalling by binding to its cellular receptor, whilst it exerts pro-inflammatory effects, such as recruitment of mononuclear cells, through trans-signalling by binding to its soluble receptor [56]. The increased IL-6 plasma levels after exercise results from muscular secretion and exerts an anti-inflammatory action [23,24]. In turn, increased PGE2 after exercise could also be considered an anti-inflammatory effector [12,54]. TNFα is considered a pro-inflammatory cytokine that increases in plasma as result of dietary functional beverage supplementation and as result of exercise in senior athletes, in any event. The measured TNFα plasma levels are similar to other studies performed with a similar beverage but in this study, no effects from exercise or supplementation were observed [13]. The excess of dietary saturated fatty acids such as palmitic acid induce peripheral inflammation [57] but polyunsaturated fatty acids such as DHA, whilst fish oil fatty acid consumption induces a peripheral anti-inflammatory response [58]. The functional beverage presents both saturated and polyunsaturated fatty acids, with predominance of unsaturated fatty acids; we detect a possible pro-inflammatory action of supplementation in young and senior athletes’ diet. In fact, dietary functional beverage supplementation increases the TNFα gene expression in PBMCs, enhancing the biosynthesis of this pro-inflammatory cytokine.

Information about the exercise effects on soluble adhesion molecule plasma levels such as sL-Selectin and sICAM 3 is scant. Resistance training does not affect the serum concentrations of the cell adhesion molecules VCAM1, ICAM1, E-selectin, sL-selectin and P-selectin [59]. On the contrary, an increase in ICAM1 after endurance training has been observed [60]; this fact could be interpreted as a protective mechanism against infections yet our results point to exercise increasing plasma sL-Selectin and sICAM3 levels whilst dietary functional beverage supplementation eliminates this exercise effect. The presence of soluble adhesion molecules in plasma indicates the degree of vascular endothelial activation or dysfunction [61], and it can reflect the status of the immune system [62]. In fact, ICAM3 is lost from neutrophils after activation [63]. It is suggested that an increase in adhesion molecules in plasma could attenuate the immune response by competing with the corresponding cell-bound adhesion molecules by cellular binding sites with leucocytes adhesion and transmigration with the endothelium response [61,64,65]; however, it has also been observed that high levels of adhesion molecules is linked to a higher cardiovascular disease risk in heart attack patients [59]. Indeed, physical fitness attenuates leukocyte-endothelial adhesion in response to exercise [66]. A dietary functional beverage abolishing the impact of exercise on sL-selectin and ICAM3 could increase leukocyte capability to adhere the endothelium but it could also reflect a reduction in leukocyte activation after exercise, attributable to dietary functional beverage consumption.

It was demonstrated that diets in rich antioxidants such as olive oil, fruits, and vegetables lower IL-6 levels in PBMCs [67]. PBMCs are primed to pro-inflammatory response after exercise, as indicated by increased NFκβ active levels in PBMCs, mainly after dietary functional beverage supplementation. PBMCs enhanced the expression of pro-inflammatory IL1β and IL-8 genes after exercise mainly in the young group after dietary functional beverage supplementation. This reinforces the pro-inflammatory impact of functional beverage consumption in young athletes partitioning exercise. Senior athletes have enhanced 15LOX gene expression after dietary functional beverage supplementation; this demonstrates higher capabilities to synthesize prostaglandins and resolvins in this situation.

In conclusion, athletic performance was not altered by dietary supplementation with a functional almond-based beverage enriched with olive oil, DHA and vitamin E for this reason it would not be necessary to supplement athletes diet with the functional beverage. Nonetheless, supplementation with a functional beverage increased erythrocyte DHA content, exercise increased plasma NEFAs (although this increase was attenuated by the supplementation with a functional beverage) and, in turn, exercise increased NFκβ activation in PBMCs. Consequently, PBMCs are primed to a pro-inflammatory response post-exercise. In the same way, exercise increased sICAM3 and sL-Selectin, but this increase was weaker after supplementation with a functional beverage. Moreover, supplementation with a functional beverage enhanced a pro-inflammatory circulatory environment in response to exercise, although this effect was less evident in senior athletes. Exercise increased PGE2 plasma levels in young supplemented athletes and in senior placebo athletes.

Conclusions of this study are limited because only five athletes of each group finished the nutritional intervention. Accordingly, the variation in human genetic background could influence the results, taking into account the small size of young and senior groups.

## Figures and Tables

**Table 1:** Subject’s anthropometric characteristics and physical activity time.

	Young	Senior
**Age (years)**	22.8 ± 3.8 ^$^	45.6 ± 1.6
**Weight (kg)**	71.0 ± 4.8	76.1 ± 2.9
**Height (cm)**	176 ± 3.8	177 ± 3.8
**Fat-free mass (%)**	9.50 ± 1.1	11.6 ± 1.0
**Body surface (m^2^)**	1.86 ± 0.05	1.93 ± 0.07
VO_2_ max (L/min*kg)	58.8 ± 2.5	50.4 ± 3.4

Statistical analysis: Two-way ANOVA, *p* < 0.05. ^$^ Differences between Young and Master groups. Results are the mean ± Standard Error Mean (SEM).

**Table 2:** Beverage fatty acid composition.

Fatty Acid	Composition
C16:0 (%)	7.62 ± 0.97
C16:1 (%)	1.20 ± 0.14
C18:0 (%)	4.24 ± 0.7
C18:1 (%)	42.5 ± 4.8
C18:2 (%)	22.9 ± 2.5
C18:3n6 (%)	0.949 ± 0.55
C18:3n3 (%)	1.21 ± 0.35
C20:0 (%)	0.368 ± 0.105
C20:1(%)	0.113 ± 0.032
C20:2 (%)	0.884 ± 0.252
C20:3 (%)	0.065 ± 0.019
C20:4n6 (%)	0.434 ± 0.124
C22:0 (%)	0.262 ± 0.075
C22:5 (%)	5.68 ± 1.62
C22:6n3 (%)	11.6 ± 3.3
Total Fatty Acids (µM)	47400 ± 9586
SFA (%)	9.90 ± 1.15
MUFA (%)	51.7 ± 5.0
PUFA (%)	38.3 ± 4.4
VITAMIN E (mg/L)	45.7 ± 27.7
POLYPHENOLS (mM)	2.85 ± 0.29

Values are the average of six samples of the functional beverage.

**Table 3:** Primer sequences and conditions.

Gene	Primer		Conditions	
18S	Fw:	5′-ATG TGA AGT CAC TGT GCC AG-3′	95 °C	10 s
	Rv:	5′-GTG TAA TCC GTC TCC ACA GA-3′	60 °C	10 s
			72 °C	12 s
TLR2	Fw:	5′-GGGTTGAAGCACTGGACAAT-3′	95 °C	10 s
	Rv:	5′-TTCTTCCTTGGAGAGGCTGA-3′	60 °C	10 s
			72 °C	15 s
TLR4	Fw:	5′-GGTCACCTTTTCTTGATTCCA-3′	95 °C	10 s
	Rv:	5′-TCAGAGGTCCATCAAACATCAC-3′	60 °C	10 s
			72 °C	15 s
TNFα	Fw:	5′-CCCAGGCAGTCAGATCATCTTCTCGGAA-3′	94 °C	10 s
	Rv:	5′-CTGGTTATCTCTCAGCTCCACGCCATT-3′	63 °C	10 s
			72 °C	15 s
NFκβ	Fw:	5′-AAA CAC TGT GAG GAT GGG ATC TG-3′	95 °C	10 s
	Rv:	5′-CGA AGC CGA CCA CCA TGT-3′	60 °C	10 s
			72 °C	15 s
COX2	Fw:	5-TTG CTG GCA GGG TTG CTG GTG GTA-3′	95 °C	10 s
	Rv:	5′-CAT CTG CCT GCT CTG GTC AAT GGA A-3′	67 °C	10 s
			72 °C	15 s
15 LOX 2	Fw:	5′-GCA TCC ACT GAT TGG ACC TT-3′	95 °C	10 s
	Rv:	5′-GCT GGC CTT GAA CTT CTG AC-3′	61 °C	10 s
			72 °C	15 s
IL1β	Fw:	5′-GGA CAG GAT ATG GAG CAA CA-3′	95 °C	10 s
	Rv:	5′-GGC AGA CTC AAA TTC CAG CT-3′	58 °C	10 s
			72 °C	15 s
5 LOX	Fw:	5′-GGG CAT GGA GAG CAA AGA AG-3	95 °C	10 s
	Rv:	5′-ACC TCG GCC GTG AAC GT-3′	59 °C	10 s
			72 °C	15 s
IL-8	Fw:	5′-GCTCTGTGTGAAGGTGCAGTTTTGCCAA-3′	95 °C	10 s
	Rv:	5′-GGCGCAGTGTGGTCCACTCTCAAT-3′	63 °C	10 s
			72 °C	15 s
IL-10	Fw:	5′-AGAACCTGAAGACCCTCAGGC-3′	95 °C	10 s
	Rv:	5′-CCACGGCCTTGCTCTTGTT-3′	60 °C	10 s
			72 °C	15 s
IL-15	Fw:	5′-CCGTGGCTTTGAGTAATGAGAATTTCGAA-3′	95 °C	10 s
	Rv:	5′-CCTGCACTGAAACAGCCCAAAATGAA-3′	60 °C	10 s
			72 °C	15 s
HSP72	Fw:	5′-CCGGCAAGGCCAACAAGATC-3′	95 °C	10 s
	Rv:	5′-CCTCCACGGCGCTCTTCATG-3′	62 °C	10 s
			72 °C	15 s

**Table 4:** Effects of Age and dietary beverage supplementation on stress test.

				ANOVA		
		Young	Senior	S	A	S × A
**Maximum Borg Index** (Borg scale)	**Control**	17.8 ± 0.49	16.2 ± 0.49			
	**Supplemented**	17.8 ± 0.48	16.6 ± 0.68			
**Time running at 90% of VO_2max_**(min)	**Control**	9.02 ± 2.99	8.84 ± 2.28			
	**Supplemented**	7.62 ± 2.59	10.0 ± 1.97			
**Initial body temperature** (°C)	**Control**	37.0 ± 0.18	37.1 ± 0.14			
	**Supplemented**	37.0 ± 0.07	37.0 ± 0.05			
**Maximum body temperature** (°C)	**Control**	39.8 ± 0.26	39.4 ± 0.15			
	**Supplemented**	39.4 ± 0.12	39.3 ± 0.20			
**Body temperature 5 min after the end** (°C)	**Control**	39.9 ± 0.23	39.5 ± 0.16			
	**Supplemented**	39.4 ± 0.15	39.3 ± 0.18			
**Maximum skin temperature** (°C)	**Control**	35.1 ± 0.21	34.7 ± 0.36			
	**Supplemented**	34.8 ± 0.19	34.8 ± 0.21			
**Skin temperature 5 min after the end** (°C)	**Control**	35.2 ± 0.24	34.5 ± 0.70			
	**Supplemented**	35.1 ± 0.15	34.7 ± 0.12			
**Maximum Lactate** (mM)	**Control**	4.08 ± 0.61	4.32 ± 0.68			
	**Supplemented**	3.86 ± 0.85	5.36 ± 0.41			
**Maximal heart rate** (beats/min)	**Control**	186 ± 4.60	175 ± 7.46			
	**Supplemented**	183 ± 6.39	175 ± 6.56			
**% of maximal heart rate during the exercise**	**Control**	97.4 ± 0.81	98.0 ±2.69			
	**Supplemented**	96.3 ± 0.68	98.0 ± 1.91			
**Water intake** (L)	**Control**	0.42 ± 0.06	0.41 ± 0.17			
	**Supplemented**	0.50 ± 0.09	0.42 ± 0.17			
**Absolute weight loss** (%)	**Control**	1.63 ± 0.08	1.41 ± 0.19			
	**Supplemented**	1.52 ± 0.17	1.66 ± 0.19			
**Wight loss without water intake** (%)	**Control**	2.21 ± 0.13	1.93 ± 0.09			
	**Supplemented**	2.22 ± 0.09	2.15 ± 0.13			
**Physiological Strain Index** [36]	**Control**	10.5 ± 0.50	9.73 ± 0.31			
	**Supplemented**	9.79 ± 0.24	9.49 ± 0.41			
**Heat Storage**(W/m^2^)	**Control**	348 ± 34.1	325 ± 19.2			
	**Supplemented**	318 ± 18.7	344 ± 47.8			

Results are the mean ± SEM. Statistical analysis: Two-way ANOVA, *p* < 0.05. S, supplementation effect; A, age effect, S × A, interaction between supplementation and age effects. * Indicates significant differences between the dietary control and functional beverage dietary supplementation plasma levels; ^$^ indicates significant differences between Young and Senior groups. When interaction exists between different statistical factors, different letters reveal significant differences.

**Table 5:** Age, dietary beverage supplementation and acute exercise effects on markers of inflammation and heat stress in plasma and inflammatory priming of peripheral blood mononuclear cells.

		Pre-Exercise		Post-Exercise		ANOVA
		Young	Senior	Young	Senior	
**sL-Selectin** (ng × mL plasma)	**Control**	106 ± 2.8 ^ab^	80.1 ± 9.7 ^ac^	146 ± 26 ^b^	89.1 ± 11.9 ^ac^	**S, S** × **E**
	**Supplemented**	77.7± 8.1 ^ac^	92.4 ± 23.1 ^ac^	61.4 ± 6.2 ^ac^	47.8 ± 13.4 ^c^	
**ICAM3** (ng of × mL plasma)	**Control**	458 ± 122 ^ade^	507 ± 55 ^acd^	670 ± 104 ^c^	705 ± 43 ^c^	**A, S, S** × **A, S** × **E**
	**Supplemented**	324 ± 65 ^ab^	604 ± 46 ^cd^	206 ±73 ^b^	618 ± 72 ^ce^	
**HSP 70** (Pg × mL of plasma)	**Control**	5.12 ± 0.31	5.01 ± 0.19	5.09 ± 0.33	4.97 ± 0.22	
	**Supplemented**	4.98 ± 0.42	5.03 ± 0.18	4.87 ± 0.47	4.74 ± 0.09	
**IL-6** (pg × mL of plasma)	**Control**	4.51 ± 0.35 ^ac^	4.27 ± 0.46 ^ac^	4.56 ± 0.40 ^ac^	5.39 ± 0.49 ^ab^	**S** × **E** × **A**
	**Supplemented**	3.54 ± 0.13 ^c^	4.87 ± 0.36 ^ac^	7.38 ± 1.21 ^b^	4.24 ± 0.36 ^ac^	
**TNFα** (pg × mL of plasma)	**Control**	627 ± 43 ^afd^	679 ± 67 ^acfd^	697 ± 52 ^acfd^	524 ± 35 ^f^	**S, S** × **E** × **A**
	**Supplemented**	831 ± 66 ^cg^	781 ± 32.4 ^abcd^	758 ± 43 ^gd^	936 ± 140 ^gb^	
**Lipoxina** (pg × mL of plasma)	**Control**	54.8 ± 11.7	67.9 ± 19.9	65.2 ± 12.5	33.5 ±10.6 #	**E**
	**Supplemented**	104 ± 29	83.4 ± 18.5	67.2 ± 9.9	36.7 ± 8.8 #	
**PGE2** (pg × mL of plasma)	**Control**	286 ± 63	398 ± 168	315 ± 71	1541± 579 #	**E**
	**Supplemented**	390 ± 112	781 ± 231	1557 ± 585 #	993 ± 476	
**PGE1** (pg × mL of plasma)	**Control**	1976 ± 168	2598 ± 735	3087± 681	5645 ± 3038	
	**Supplemented**	3593 ± 995	3273 ± 1204	2465 ± 427	3947 ± 980	
**NFκβ** (U.A.)	**Control**	2628 ± 140	2720 ± 249	2877 ±135	2889 ±194	**E**
	**Supplemented**	2437 ± 210	2730 ± 210	3099 ± 343 #	3400 ± 167 #	

Results are the mean ± SEM. Statistical analysis: Three-way ANOVA, *p* < 0.05. S, supplementation effect; A, age effect, E, exercise effect, S × A, interaction between supplementation and age effects, S × E, interaction between supplementation and exercise effects, E × A, interaction between exercise and age effects, A × E × S, interaction between three factors effects. S, A, E, S × A, E × A, S × E or A × E × S indicates a significant effect of each statistical factor. * Indicates significant differences between the dietary control and functional beverage dietary supplementation plasma levels; # indicates significant differences between pre-exercise and post-exercise plasma levels; ^$^ indicates significant differences between Young and Senior groups. When interaction exists between different statistical factors, different letters reveal significant differences.

**Table 6:** Effects of dietary functional beverage supplementation, exercise and age on the expression of inflammatory and related genes.

		Pre-Exercise		Post-Exercise		ANOVA
		Young	Senior	Young	Senior	
**TLR2**	**Control**	1.00 ± 0.28	1.59 ± 0.56	0.99 ± 0.29	0.77 ± 0.10	**E** × **A**
	**Supplemented**	1.13 ± 0.37	1.01 ± 0.26	2.51 ± 1.11	0.84 ± 0.13	
**TLR4**	**Control**	1.00 ± 0.24	1.18 ± 0.41	1.25 ± 0.47	0.86 ± 0.12	
	**Supplemented**	1.46 ± 0.55	1.15 ± 0.29	2.62 ± 1.19	0.84 ± 0.11	
**NFκβ**	**Control**	1.00 ± 0.21	1.12 ± 0.34	1.11 ± 0.30	0.89 ± 0.12	
	**Supplemented**	1.75 ± 0.79	1.74 ± 0.67	1.39 ± 0.46	0.87 ± 0.09	
**COX2**	**Control**	1.00 ± 0.09	1.07 ± 0.17	1.39 ± 0.38	1.50 ± 0.41	**A** (0.094)
	**Supplemented**	2.31 ± 1.09	1.36 ± 0.36	3.99 ± 1.96	1.20 ± 0.24	**S** × **A** (0.066)
**5LOX**	**Control**	1.00 ± 0.19	1.21 ± 0.40	1.14 ± 0.28	1.10 ± 0.28	
	**Supplemented**	1.15 ± 0.34	1.57 ± 0.53	3.59 ± 1.97	0.96 ± 0.13	
**15LOX2**	**Control**	1.00 ± 0.33	1.63 ± 0.45	0.96 ± 0.22	2.14 ± 0.77	**A** (0.035)
	**Supplemented**	2.14 ± 0.77	6.86 ± 4.21 *^,$^	1.13 ± 0.36	7.65 ± 2.52 *^,$^	**S** (0.048)
**IL1β**	**Control**	1.00 ± 0.14 ^a^	0.99 ± 0.17 ^a^	1.17 ± 0.25 ^a^	1.68 ± 0.73 ^a^	**S** (0.084)
	**Supplemented**	1.92 ± 0.59 ^a^	1.10 ± 0.22 ^a^	3.86 ± 1.54 ^b^	1.28 ± 0.34 ^a^	**S** × **A** (0.043)
**IL-8**	**Control**	1.00 ± 0.28 ^a^	0.92 ± 0.20 ^a^	0.74 ± 0.11 ^a^	1.74 ± 0.75 ^a^	**S** × **A** (0.029)
	**Supplemented**	2.39 ± 1.19 ^ab^	0.94 ± 0.29 ^a^	4.10 ± 2.48 ^b^	0.73 ± 0.11 ^a^	
**TNFα**	**Control**	1.00 ± 0.11	1.06 ± 0.19	1.07 ± 0.21	1.19 ± 0.39	**S** (0.032)
	**Supplemented**	1.16 ± 0.21	1.21 ± 0.42	1.72 ± 0.58	2.37 ± 0.59	
**IL-10**	**Control**	1.00 ± 0.23	1.56 ± 0.60	1.45 ± 0.59	1.15 ± 0.37	
	**Supplemented**	1.71 ± 0.75	1.51 ± 0.45	3.41 ± 1.66	1.54 ± 0.57	
**IL-15**	**Control**	1.00 ± 0.25	0.90 ± 0.23	0.91 ± 0.17	1.35 ± 0.52	
	**Supplemented**	1.18 ± 0.35	1.16 ± 0.35	1.97 ± 0.89	0.84 ± 0.15	
**HSP72**	**Control**	1.00 ± 0.28	1.07 ± 0.31	1.01 ± 0.31	0.81 ± 0.13	
	**Supplemented**	2.48 ± 1.39	1.23 ± 0.49	2.53 ± 1.17	0.79 ± 0.12	

Results are the mean ± SEM. Statistical analysis: Three-way ANOVA, *p* < 0.1. S, supplementation effect; A, age effect, E, exercise effect, S × A, interaction between supplementation and age effects, S × E, interaction between supplementation and exercise effects, E × A, interaction between exercise and age effects, A × E × S, effects of interaction between three factors. S, A, E, S × A, E × A, S × E or A × E × S indicates a significant effect of each statistical factor. * Indicates significant differences between the dietary control and functional beverage dietary supplementation; # indicates significant differences between pre-exercise and post-exercise; ^$^ indicates significant differences between Young and Senior groups. Where interaction exists between different statistical factors, different letters reveal significant differences.

## References

[1] Milbury P.E., Chen C.Y., Dolnikowski G.G., Blumberg J.B. (2006). Determination of flavonoids and phenolics and their distribution in almonds. J. Agric. Food Chem..

[2] Li N., Jia X., Chen C.Y., Blumberg J.B., Song Y., Zhang W., Zhang X., Ma G., Chen J. (2007). Almond consumption reduces oxidative DNA damage and lipid peroxidation in male smokers. J. Nutr..

[3] Liu R.H. (2004). Potential synergy of phytochemicals in cancer prevention: Mechanism of action. J. Nutr..

[4] Jenkins D.J., Kendall C.W., Marchie A., Josse A.R., Nguyen T.H., Faulkner D.A., Lapsley K.G., Blumberg J. (2008). Almonds reduce biomarkers of lipid peroxidation in older hyperlipidemic subjects. J. Nutr..

[5] Tresserra-Rimbau A., Medina-Remon A., Perez-Jimenez J., Martinez-Gonzalez M.A., Covas M.I., Corella D., Salas-Salvado J., Gomez-Gracia E., Lapetra J., Aros F. (2013). Dietary intake and major food sources of polyphenols in a Spanish population at high cardiovascular risk: The predimed study. Nutr. Metab. Cardiovasc. Dis..

[6] Guasch-Ferre M., Hu F.B., Martinez-Gonzalez M.A., Fito M., Bullo M., Estruch R., Ros E., Corella D., Recondo J., Gomez-Gracia E. (2014). Olive oil intake and risk of cardiovascular disease and mortality in the predimed study. BMC Med..

[7] Urpi-Sarda M., Casas R., Chiva-Blanch G., Romero-Mamani E.S., Valderas-Martinez P., Arranz S., Andres-Lacueva C., Llorach R., Medina-Remon A., Lamuela-Raventos R.M. (2012). Virgin olive oil and nuts as key foods of the mediterranean diet effects on inflammatory biomakers related to atherosclerosis. Pharmacol. Res..

[8] Martinez-Gonzalez M.A., Toledo E., Aros F., Fiol M., Corella D., Salas-Salvado J., Ros E., Covas M.I., Fernandez-Crehuet J., Lapetra J. (2014). Extravirgin olive oil consumption reduces risk of atrial fibrillation: The PREDIMED (prevencion con dieta mediterranea) trial. Circulation.

[9] Medina-Remon A., Tresserra-Rimbau A., Pons A., Tur J.A., Martorell M., Ros E., Buil-Cosiales P., Sacanella E., Covas M.I., Corella D. (2015). Effects of total dietary polyphenols on plasma nitric oxide and blood pressure in a high cardiovascular risk cohort. The predimed randomized trial. Nutr. Metab. Cardiovasc. Dis..

[10] Petroczi A., Naughton D.P., Mazanov J., Holloway A., Bingham J. (2007). Performance enhancement with supplements: Incongruence between rationale and practice. J. Int. Soc. Sports Nutr..

[11] Mickleborough T.D. (2013). Omega-3 polyunsaturated fatty acids in physical performance optimization. Int. J. Sport Nutr. Exerc. Metab..

[12] Martorell M., Capo X., Sureda A., Batle J.M., Llompart I., Argelich E., Tur J.A., Pons A. (2014). Effect of DHA on plasma fatty acid availability and oxidative stress during training season and football exercise. Food Funct..

[13] Capo X., Martorell M., Llompart I., Sureda A., Tur J.A., Pons A. (2014). Docosahexanoic acid diet supplementation attenuates the peripheral mononuclear cell inflammatory response to exercise following LPS activation. Cytokine.

[14] Capo X., Martorell M., Sureda A., Batle J.M., Tur J.A., Pons A. (2016). Docosahexaenoic diet supplementation, exercise and temperature affect cytokine production by lipopolysaccharide-stimulated mononuclear cells. J. Physiol. Biochem..

[15] Garcia J.J., Bote E., Hinchado M.D., Ortega E. (2011). A single session of intense exercise improves the inflammatory response in healthy sedentary women. J. Physiol. Biochem..

[16] Pedersen B.K., Rohde T., Zacho M. (1996). Immunity in athletes. J. Sports Med. Phys. Fit..

[17] Nieman D.C., Pedersen B.K. (1999). Exercise and immune function. Recent developments. Sports Med..

[18] Ozen A.E., Pons A., Tur J.A. (2012). Worldwide consumption of functional foods: A systematic review. Nutr. Rev..

[19] Sureda A., Tauler P., Aguilo A., Cases N., Fuentespina E., Cordova A., Tur J.A., Pons A. (2005). Relation between oxidative stress markers and antioxidant endogenous defences during exhaustive exercise. Free Radic. Res..

[20] Mestre-Alfaro A., Ferrer M.D., Sureda A., Tauler P., Martinez E., Bibiloni M.M., Micol V., Tur J.A., Pons A. (2011). Phytoestrogens enhance antioxidant enzymes after swimming exercise and modulate sex hormone plasma levels in female swimmers. Eur. J. Appl. Physiol..

[21] Watson T.A., MacDonald-Wicks L.K., Garg M.L. (2005). Oxidative stress and antioxidants in athletes undertaking regular exercise training. Int. J. Sport Nutr. Exerc. Metab..

[22] Volpe S.L. (2007). Micronutrient requirements for athletes. Clin. Sports Med..

[23] Pedersen B.K., Febbraio M.A. (2008). Muscle as an endocrine organ: Focus on muscle-derived Interleukin-6. Physiol. Rev..

[24] Walsh N.P., Gleeson M., Shephard R.J., Gleeson M., Woods J.A., Bishop N.C., Fleshner M., Green C., Pedersen B.K., Hoffman-Goetz L. (2011). Position statement part one: Immune function and exercise. Exerc. Immunol. Rev..

[25] Pedersen B.K., Saltin B. (2006). Evidence for prescribing exercise as therapy in chronic disease. Scand. J. Med. Sci. Sports.

[26] Calbet J.A. (2012). Ageing, exercise and cardiovascular health: Good and bad news. J. Physiol..

[27] Pawelec G., Goldeck D., Derhovanessian E. (2014). Inflammation, ageing and chronic disease. Curr. Opin. Immunol..

[28] Bailey D.M., McEneny J., Mathieu-Costello O., Henry R.R., James P.E., McCord J.M., Pietri S., Young I.S., Richardson R.S. (2010). Sedentary aging increases resting and exercise-induced intramuscular free radical formation. J. Appl. Physiol..

[29] Meydani M. (1992). Vitamin E requirement in relation to dietary fish oil and oxidative stress in elderly. Exs.

[30] Molfino A., Gioia G., Rossi Fanelli F., Muscaritoli M. (2014). The role for dietary omega-3 fatty acids supplementation in older adults. Nutrients.

[31] McFarlin B.K., Mitchell J.B. (2003). Exercise in hot and cold environments: Differential effects on leukocyte number and NK cell activity. Aviat. Space Environ. Med..

[32] Borg G. (1970). Perceived exertion as an indicator of somatic stress. Scand. J. Rehabil. Med..

[33] Capo X., Martorell M., Sureda A., Llompart I., Tur J.A., Pons A. (2015). Diet supplementation with DHA-enriched food in football players during training season enhances the mitochondrial antioxidant capabilities in blood mononuclear cells. Eur. J. Nutr..

[34] Martorell M., Capo X., Bibiloni M.M., Sureda A., Mestre-Alfaro A., Batle J.M., Llompart I., Tur J.A., Pons A. (2015). Docosahexaenoic acid supplementation promotes erythrocyte antioxidant defense and reduces protein nitrosative damage in male athletes. Lipids.

[35] Kubiliene L., Laugaliene V., Pavilonis A., Maruska A., Majiene D., Barcauskaite K., Kubilius R., Kasparaviciene G., Savickas A. (2015). Alternative preparation of propolis extracts: Comparison of their composition and biological activities. BMC Complement. Altern. Med..

[36] Tepsic J., Vucic V., Arsic A., Blazencic-Mladenovic V., Mazic S., Glibetic M. (2009). Plasma and erythrocyte phospholipid fatty acid profile in professional basketball and football players. Eur. J. Appl. Physiol..

[37] Rodriguez N.R., DiMarco N.M., Langley S. (2009). Position of the American dietetic association, dietitians of Canada, and the American college of sports medicine: Nutrition and athletic performance. J. Am. Diet. Assoc..

[38] Neubauer O., Yfanti C., Lamprecht M. (2015). Antioxidants in athlete’s basic nutrition: Considerations towards a guideline for the intake of vitamin C and vitamine. Antioxidants in Sport Nutrition.

[39] Rangel-Huerta O.D., Aguilera C.M., Martin M.V., Soto M.J., Rico M.C., Vallejo F., Tomas-Barberan F., Perez-de-la-Cruz A.J., Gil A., Mesa M.D. (2015). Normal or high polyphenol concentration in orange juice affects antioxidant activity, blood pressure, and body weight in obese or overweight adults. J. Nutr..

[40] Bentley D.J., Ackerman J., Clifford T., Slattery K.S., Lamprecht M. (2015). Acute and chronic effects of antioxidant supplementation on exercise performance. Antioxidants in Sport Nutrition.

[41] Fleming J., Sharman M.J., Avery N.G., Love D.M., Gomez A.L., Scheett T.P., Kraemer W.J., Volek J.S. (2003). Endurance capacity and high-intensity exercise performance responses to a high fat diet. Int. J. Sport Nutr. Exerc. Metab..

[42] Rodacki C.L., Rodacki A.L., Pereira G., Naliwaiko K., Coelho I., Pequito D., Fernandes L.C. (2012). Fish-oil supplementation enhances the effects of strength training in elderly women. Am. J. Clin. Nutr..

[43] Lembke P., Capodice J., Hebert K., Swenson T. (2014). Influence of omega-3 (*n*-3) index on performance and wellbeing in young adults after heavy eccentric exercise. J. Sports Sci. Med..

[44] Sureda A., Mestre-Alfaro A., Banquells M., Riera J., Drobnic F., Camps J., Joven J., Tur J.A., Pons A. (2015). Exercise in a hot environment influences plasma anti-inflammatory and antioxidant status in well-trained athletes. J. Therm. Biol..

[45] Basu A., Devaraj S., Jialal I. (2006). Dietary factors that promote or retard inflammation. Arterioscler. Thromb. Vasc. Boil..

[46] Huang S., Rutkowsky J.M., Snodgrass R.G., Ono-Moore K.D., Schneider D.A., Newman J.W., Adams S.H., Hwang D.H. (2012). Saturated fatty acids activate TLR-mediated proinflammatory signaling pathways. J. Lipid Res..

[47] Rajaram S., Connell K.M., Sabate J. (2010). Effect of almond-enriched high-monounsaturated fat diet on selected markers of inflammation: A randomised, controlled, crossover study. Br. J. Nutr..

[48] Pedersen B.K., Hoffman-Goetz L. (2000). Exercise and the immune system: Regulation, integration, and adaptation. Physiol. Rev..

[49] Peake J.M., Suzuki K., Hordern M., Wilson G., Nosaka K., Coombes J.S. (2005). Plasma cytokine changes in relation to exercise intensity and muscle damage. Eur. J. Appl. Physiol..

[50] Pararasa C., Ikwuobe J., Shigdar S., Boukouvalas A., Nabney I.T., Brown J.E., Devitt A., Bailey C.J., Bennett S.J., Griffiths H.R. (2016). Age-associated changes in long-chain fatty acid profile during healthy aging promote pro-inflammatory monocyte polarization via ppargamma. Aging Cell.

[51] Pahl H.L. (1999). Activators and target genes of Rel/NF-kappab transcription factors. Oncogene.

[52] Capo X., Martorell M., Sureda A., Tur J.A., Pons A. (2015). Effects of docosahexaenoic supplementation and in vitro vitamin C on the oxidative and inflammatory neutrophil response to activation. Oxid. Med. Cell. Longev..

[53] Markworth J.F., Vella L., Lingard B.S., Tull D.L., Rupasinghe T.W., Sinclair A.J., Maddipati K.R., Cameron-Smith D. (2013). Human inflammatory and resolving lipid mediator responses to resistance exercise and ibuprofen treatment. Am. J. Physiol. Regul. Integr. Comp. Physiol..

[54] Capo X., Martorell M., Sureda A., Tur J.A., Pons A. (2016). Effects of dietary docosahexaenoic, training and acute exercise on lipid mediators. J. Int. Soc. Sports Nutr..

[55] Hirose L., Nosaka K., Newton M., Laveder A., Kano M., Peake J., Suzuki K. (2004). Changes in inflammatory mediators following eccentric exercise of the elbow flexors. Exerc. Immunol. Rev..

[56] Scheller J., Rose-John S. (2006). Interleukin-6 and its receptor: From bench to bedside. Med. Microbial. Immunol..

[57] Wu D., Liu J., Pang X., Wang S., Zhao J., Zhang X., Feng L. (2014). Palmitic acid exerts pro-inflammatory effects on vascular smooth muscle cells by inducing the expression of c-reactive protein, inducible nitric oxide synthase and tumor necrosis factor-alpha. Int. J. Mol. Med..

[58] Calder P.C. (2006). *N*-3 polyunsaturated fatty acids, inflammation, and inflammatory diseases. Am. J. Clin. Nutr..

[59] Petridou A., Chatzinikolaou A., Fatouros I., Mastorakos G., Mitrakou A., Chandrinou H., Papassotiriou I., Mougios V. (2007). Resistance exercise does not affect the serum concentrations of cell adhesion molecules. Br. J. Sports Med..

[60] Baum M., Liesen H., Enneper J. (1994). Leucocytes, lymphocytes, activation parameters and cell adhesion molecules in middle-distance runners under different training conditions. Int. J. Sports Med..

[61] Carlos T.M., Harlan J.M. (1994). Leukocyte-endothelial adhesion molecules. Blood.

[62] Akimoto T., Furudate M., Saitoh M., Sugiura K., Waku T., Akama T., Kono I. (2002). Increased plasma concentrations of intercellular adhesion molecule-1 after strenuous exercise associated with muscle damage. Eur. J. Appl. Physiol..

[63] Del Pozo M.A., Pulido R., Munoz C., Alvarez V., Humbria A., Campanero M.R., Sanchez-Madrid F. (1994). Regulation of ICAM-3 (CD50) membrane expression on human neutrophils through a proteolytic shedding mechanism. Eur. J. Immunol..

[64] Smith L.L., Anwar A., Fragen M., Rananto C., Johnson R., Holbert D. (2000). Cytokines and cell adhesion molecules associated with high-intensity eccentric exercise. Eur. J. Appl. Physiol..

[65] Nielsen H.G., Lyberg T. (2004). Long-distance running modulates the expression of leucocyte and endothelial adhesion molecules. Scand. J. Immunol..

[66] Mills P.J., Hong S., Redwine L., Carter S.M., Chiu A., Ziegler M.G., Dimsdale J.E., Maisel A.S. (2006). Physical fitness attenuates leukocyte-endothelial adhesion in response to acute exercise. J. Appl. Physiol..

[67] Munoz A., Costa M. (2013). Nutritionally mediated oxidative stress and inflammation. Oxid. Med. Cell. Longev..

